# Rhythm‐Control History in AFIRE: What Can We Conclude?

**DOI:** 10.1002/joa3.70361

**Published:** 2026-05-14

**Authors:** Aytuğ Al, Şuara İrem Al, Bülent Görenek

**Affiliations:** ^1^ Department of Cardiology Yunus Emre State Hospital Eskişehir Turkey; ^2^ Department of Cardiology, Faculty of Medicine Eskisehir Osmangazi University Eskisehir Turkey

We read with interest the AFIRE subanalysis by Wakatsuki et al. examining “rhythm‐control history” and clinical outcomes in atrial fibrillation with stable coronary artery disease [1]. The apparent benefit in unadjusted analyses did not persist after multivariable adjustment and propensity score matching.

Given that rhythm‐control decisions were physician‐directed and post‐treatment rhythm status was not assessed, this analysis primarily reflects treatment history rather than rhythm‐control success, limiting causal interpretation. Receiving rhythm‐control therapy does not establish that rhythm control was achieved or sustained. In this setting, “history” should not be interpreted as “successful rhythm control”. Moreover, rhythm‐control history was more common in younger, less comorbid patients (e.g., higher rates of paroxysmal AF, less heart failure, with lower CHADS_2_/CHA_2_DS_2_‐VASc scores), suggesting patient selection. We offer several suggestions to clarify and strengthen the interpretation.

First, “history of rhythm control” is a broad and heterogeneous exposure. Reporting baseline characteristics and outcomes separately for antiarrhythmic drug therapy only, catheter ablation only, and both would suggest whether one component drives the findings [2]. Clarifying whether electrical cardioversion—a widely used, readily available, and cost‐effective rhythm‐control approach—was captured in AFIRE and how it was classified within the “rhythm‐control history” definition would be valuable. A history of electrical cardioversion may reflect a previous rhythm‐restoration attempt rather than sustained rhythm control and may identify patients with persistent AF and potentially a higher AF burden. Repeated cardioversions, in particular, may indicate recurrent AF. Whether such patients are included in the rhythm‐control history group may therefore affect group composition and the interpretation of the study findings. In addition, stratifying by the time since the last rhythm‐control intervention could assess whether timing influences outcomes [3].

Second, if practical proxies of rhythm status exist (e.g., documented recurrences, cardioversions, repeat catheter ablation, or rhythm status on follow‐up ECGs), a brief sensitivity analysis using these proxies would add valuable context.

Third, in a cohort defined by stable coronary artery disease, accounting for CAD burden is central to interpretation. However, the propensity/adjusted models primarily emphasize overall atherosclerotic comorbidity risk (e.g., age, CHF, AF type, and CHADS_2_) rather than CAD burden itself. Adding available CAD‐related variables (prior myocardial infarction, PCI, or CABG) to the adjustment strategy could strengthen interpretation and reduce residual confounding.

Finally, as results are presented separately for rivaroxaban monotherapy and combination therapy in AFIRE [4], a formal interaction test between rhythm‐control history and antithrombotic strategy would be helpful. As a minor point, we noted that a hazard ratio and its confidence interval in table 3 may be inconsistent, and we would appreciate clarification if this is due to a typographical error [1].

We congratulate the authors on this informative subanalysis and hope these clarifications will help readers interpret the findings and guide future analyses in this clinically important population.

## Conflicts of Interest

The authors declare no conflicts of interest.

## Data Availability

Data sharing not applicable to this article as no datasets were generated or analysed during the current study.
